# Statistical Optimization of Process Parameters by Central Composite Design (CCD) for an Enhanced Production of L-asparaginase by *Myroides gitamensis* BSH-3, a Novel Species

**Published:** 2019

**Authors:** VSSL Prasad Talluri, Sri Santhi Lanka, V Rajagopal Saladi

**Affiliations:** 1. Department of Biotechnology, College of Natural and Computational Science, University of Gondar, Gondar, Ethiopia; 2. Department of Biotechnology, GITAM Institute of Science, GITAM University, Visakhapatnam- 530 045, Andhra Pradesh, India; 3. Department of Biotechnology, University of Chemistry and Technology Prague, Technicka 5, CZ 16628 Prague 6, Czech Republic

**Keywords:** Biotransformation, Bran, Environmental pollution, Fermentation, L-asparaginase, *Myroides gitamensis*, Wheat

## Abstract

**Background:**

The present study focused on the production of L-asparaginase using Solid State Fermentation (SSF) by *Myroides gitamensis.*

**Methods:**

Initially, five significant parameters (Carbon source; Nitrogen source, temperature, pH and incubation period) were identified that affect the production process of L-asparaginase using Classical One Factor at a Time (OFAT) optimization. An optimized L-asparaginase specific activity obtained by OFAT was recorded as 85.7 *IU*. Central Composite Design (CCD) was also employed successively to optimize the multiple parameters at a time and their results were compared.

**Results:**

Maximum L-asparaginase enzyme specific activity obtained by CCD method was 295.6 *IU* under the hold values of carbon source (wheat bran) 12 *g/L*, nitrogen source (yeast extract) 7 *g/L*, temperature 37°*C*, pH=7.5 and incubation period 47 *hr*. Upon validation, the obtained results proved that there was a good relation existing between the experimental and the predicted model (p<0.05). L-asparaginase activity was enhanced in statistical method up to 3.4 folds compared to that of classical method.

**Conclusion:**

Utilization of wheat bran as a low cost carbon source in SSF for the production of L-asparaginase enzyme makes the process economical and in turn reduces the environmental pollution by biotransformation to commercially useful bio product.

## Introduction

L-asparaginase (EC. No.- 3.5.1.1) catalyses the conversion of L-asparagine into L-aspartic acid and ammonia. L-asparaginase has been widely used in chemotherapy in the field of medicine. L-asparaginase has been proved as a potential enzyme for the treatment of Acute Lymphocytic Leukaemia (ALL) and Lymphosarcoma Cancer 1. The therapeutic property of L-asparaginase seeks commercial importance, showing significant role in modern oncology 2. It acts as a model enzyme in the field of drug discovery and also acts as biosensor for leukemia 3.

From the available literature, it was evident that if L-asparaginase is given to cancer patients, there would be enormous reduction in L-asparagine level from its pool 4. L-asparaginase also plays an important role in food industry by producing an acrylamide free food 5.

Unlike normal cells, the malignant cells require large amount of L-asparagine for protein synthesis and cell division, as L-asparaginase catalyzes the deamination of L-asparagine into L-aspartic acid and ammonia, hence L-asparagine levels in the cancer cells decrease drastically, thereby it leads to the decrease in protein synthesis and cell division in tumor cells. In normal cells, oxaloacetate is converted to aspartate by transamination which further converts into L-asparagine by asparagine synthetase which converts the carboxy group to amide group in the side chain. The gene which codes for asparagine synthetase was located on chromosome number No. 7 (7q21.4) in humans. Tumor cells are unable to produce L-asparagine due to the lack of asparagine synthetase, hence they should obtain this amino acid from the blood circulation. In murine leukemia cell lines, protein synthesis is repressed in the absence of L-asparagine, which results in cell cycle arrest and apoptosis 6.

Therapeutic enzymes act on their target site with a high affinity and great specificity and they won’t exhibit any side effects. At present, L-asparaginase is available in the market with different brand names, such as ERWINASE, CLOLAR, LEUKINE, ONCASPAR, ARRANON, KIDROLASE and ELSPAR.

Microbial enzymes are preferred over animal or plant enzymes due to their consistency, cost-effective production, ease of process modification and optimization. These enzymes show higher stability than the enzymes that are derived from animals and plants 7. Microbial enzymes provide a greater diversity of catalytic activities and wide range of specificities and stability to perform their biochemical reactions.

Several agro manufacturing byproducts such as rice bran, wheat bran, green gram bran, coconut cake, groundnut cake, soya meal, *etc*. have been used for the production of different microbial enzymes. Among all agro industrial by-products, wheat bran is the most commonly used substrate as it has been reported to have nourishing components required for the growth of microorganisms 8. The composition of wheat bran contains different soluble sugars like glucose, arabinose, galactose, xylose, *etc*. These soluble sugars are useful for the initiation of cell growth and replication of microbes 9. Simultaneously, wheat bran has higher amounts of protein and low lignin content as compared to other substrates. Therefore, wheat bran is preferred as the better substrate for the production of microbial enzymes.

Classical One Factor At a Time (OFAT) design facilitates optimizing the process parameters individually and the designs obtained are quite useful for awaking the optimized conditions due to which the principal parameters either can be considered or eliminated for further optimization processes 10. Thus, optimum parameters of the entire process have been determined using statistical tools such as regression of full factorial to obtain a statistical model. The entire optimization process involved in the L-asparaginase enzyme production, usually involves fitting of data to a polynomial equation, using multiple regression analysis and one way ANOVA. Hence, various interactions of the experimental design and predicted models are needed for an efficient production of L-asparaginase with an enhanced yield through Solid State Fermentation system (SSF).

Currently, the application of both Classical One Factor At a Time (OFAT) and Central Composite Design (CCD) for the optimization of process parameters were the main focus, which was taken into consideration for the enhanced production of L-asparaginase by supplementing wheat bran as a carbon source for the *Myroides gitamensis (M. gitamensis)*. The bioprocess optimization was done by stepwise experimental strategy *i.e*.,: (1) screening the most significant factors affecting enzyme production using a Classical One Factor At a Time (OFAT) method, (2) optimization of the significant screened parameters and generating a surface interaction plot showing the relationship between optimized parameters and L-asparaginase production by the application of Central Composite Design (CCD) and (3) validation of the model for the entire production process.

## Materials and Methods

### Microorganism

*Myroides* strain BSH-3 was isolated from slaughter house soil samples, Visakhapatnam, India and identified as *M. gitamensis* (GenBank Accession Number HF571338) on the basis of morphological, biochemical and 16S ribosomal RNA gene sequence 11 and submitted in microbial type culture collection (MTCC 11601), Bangkok culture collection (BCC 64301) and microbial culture collection (MCC 2182). The isolated pure strain of *M. gitamensis* was maintained on nutrient agar slants and incubated at 37 °*C* for 24 *hr*. After 24 *hr* of incubation, the active culture was used for production process.

### Media composition and culture conditions

Firstly, *M. gitamensis* BSH-3 was cultured using basal medium containing (*g/L*): glucose-2.0, L-asparagine-10.0, KH_2_PO_4_-1.52, KCl-0.52, MgSO_4_. 7H_2_O-0.52, CuNO_3_.3H_2_O-trace, ZnSO_4_.7H_2_O-trace and FeSO_4_. 7H_2_O-trace 12. The pH was then adjusted to 7.0 using 0.1 *N* NaOH and 0.1 *N* HCl before autoclaving. One *ml* of BSH-3 strain overnight culture (1×10^8^ *cells/ml*) was transferred aseptically into an Erlenmeyer flask (250 *ml*) containing 50 *ml* of basal medium and then incubated at 37°*C*, on a rotary shaker at 120 *pm* for 24 *hr*.

### Determination of bacterial growth rate

Absorbance (A_600_) of cultured broth was measured for every 4 *hr* up to 48 *hr* for stable growth using UV-VIS spectrophotometer (Shimadzu U-800). A basal medium lacking the inoculum was also maintained as a control.

### Estimation of L-asparaginase activity

L-asparaginase activity was estimated according to the method of Mashburn and Wriston 13. In this assay, the reaction mixture consisting of 100 *μl* enzyme extract, 200 *μl* 0.05 *M* Tris-Hcl buffer (pH=8.6) and 1.7 *ml* of 0.01 *M* L-asparagine was incubated for 10 *min* at 37 °*C*. The rate of hydrolysis of L-asparagine was determined by measuring the released ammonia using Nessler’s reagent.

The reaction was stopped by the addition of 500 *μl* of 1.5 *M* TCA. Then, the reaction mixture was centrifuged at 1000 *rcf* at 4 °*C* for 10 *min* and then 0.5 *ml* of supernatant was diluted to 7 *ml* with distilled water and 1 *ml* of Nessler’s reagent was added. The reaction mixture was incubated in boiling water bath for 10 *min* and then cooled. Later, the absorbance (A_500_) was recorded using UV-Vis spectrophotometer (ShimazduU-800) followed by protein estimation according to method of Lowry’s 14. The ammonia liberated was entrapped from a curve derived with ammonia sulphate as standard curve and expressed in IU.

### Classical optimization

After the identification of significant parameters, the optimization process was done by Classical One Factor At a Time (OFAT) method by varying single factor only and keeping the remaining factors constant 15. The optimal level of substrate (wheat bran) was studied by varying the concentration (2–14 *g/L*). Different nitrogen sources (Yeast extract, beef extract, peptone, tryptone, malt extract and urea) were used over the range of 0.5% and carbon sources (arabinose, fructose, dextrose, lactose, maltose, starch and sucrose) at a final concentration of 0.2%. Optimum pH, temperature and incubation period were determined by studying in the range of 5 to 9, 25 to 55°*C* and 24 to 48 *hr*, respectively. Samples were drawn continuously at 24 *hr* interval and then the enzyme and protein assay was carried out to calculate the enzyme specific activity.

### Central Composite Design based optimization (CCD)

A five level RSM based CCD was employed for optimization process parameters of L-asparaginase production from *M. gitamenesis*. Based on the observations with classical OFAT experiments, five parameters were selected in the range, pH (6.5–8.5), temperature (36–38*°C*), wheat bran (11–13 *g/L*), incubation time (47–49 *hr*) and yeast extract (5–7 *g/L*). All the processes were done in triplicates and represented in coded terms as the lowest, central and the highest level of five variables indicating as −1, 0 and +1, respectively (Table 1). The observations resulted with response surface methodology were recorded and interpreted with their respective possible surface interaction plots.

**Table 1.** Coded and actual levels of the independent variables for the design of CCD experiment

**Independent variables**	**Symbols**	**Coded Levels**		
		**−1**	**0**	**+1**
**Yeast extract (*g*)**	A	5	6	7
**pH**	B	6.5	7.5	8.5
**Temperature (°*C*)**	C	37	37	38
**Wheat bran (*g*)**	D	11	12	13
**Incubation time (*hr*)**	E	47	48	49

### Purification of crude enzyme

The 36 *hr* culture broth was centrifuged at 11,200 *rcf* for 30 *min* at 4 °*C* using plastocrafts refrigerated centrifuge. The supernatant was collected and stored at 4 °*C* for further analysis. Ammonium sulfate precipitation was performed according to the method of Charles *et al* 16. The active ammonium sulfate fractionation (3.0 *ml*) was loaded onto a column of sephadex G-200 17.

SDS-PAGE was carried out as described by Laemmli 18 using 15 *cm* 10% acryl amide gel. Cathode and anode were at the top and bottom of the gel, respectively. About 30 *μl* of crude, ammonium sulfate precipitation fractionation, gel filtration fractions and markers (1 *mg/ml*) were loaded into each well and electrophoresed at constant voltage of 100 *V* of electric field at 4 °*C* until the bromophenol blue moved to the bottom of the gel. Bovine serum albumin, 66 *kDa*; ovalbumin, 43 *kDa*; carbonic anhydrase, 20.1 *kDa*; cytochrome-C, 12 *kDa* were used as markers.

### Statistical analysis

The observations obtained by both Classical One Factor At a Time (OFAT) and Central Composite Design (CCD) in the present investigation were analyzed using Minitab (version 16.0). Statistical analysis of the observations was evaluated through analysis of variance (ANOVA) and the regression equation was generated along with the predicted responses. The response surface was expressed at the following second-order polynomial equation represented as follows:

$$\overset{\text{k}}{Y}=\beta_{0}+\sum \overset{\text{k}}{\beta_{1}}X_{i}+\sum_{\text{i}=1}\beta_{ii}X_{i}^{2}+\sum_{\text{i}=1}\sum \beta_{ij}X_{i}X_{j}$$

Where Y is the predicted response; K is the number of factor variables; *β*_0_ is the model constant; *β_i_* is the linear coefficient; *β_ii_* is the quadratic coefficient; *β_ij_* is the interaction coefficient. “X_i_” is the factor variable in its coded form.

## Results

### Classical optimization

**Figure 1.**
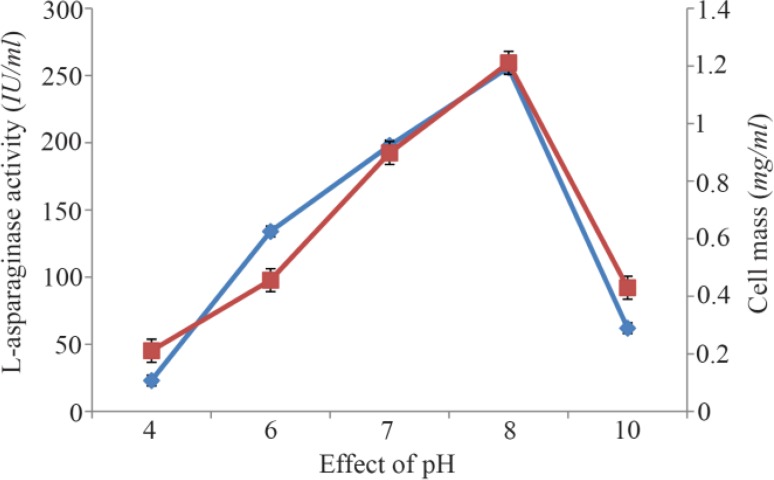
Effect of pH on growth and L-asparaginase production.

**Figure 2.**
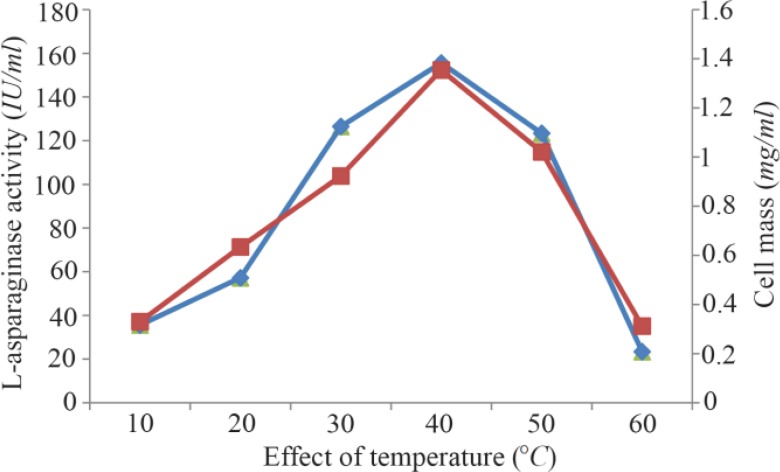
Effect of temperature on growth and L-asparaginase production.

**Figure 3.**
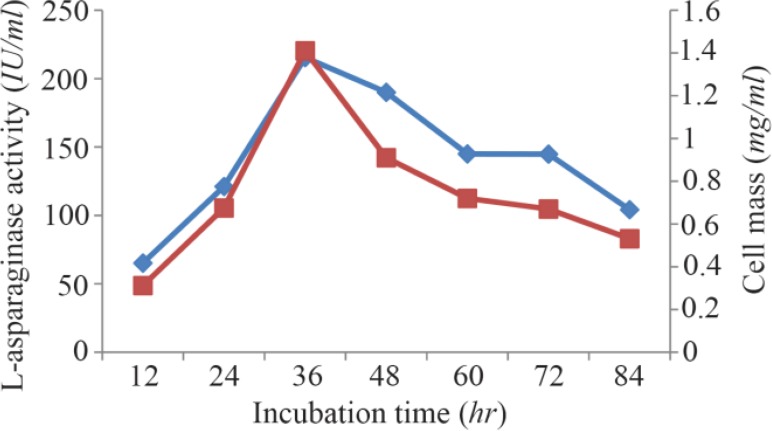
Effect of incubation time on growth and L-asparaginase production.

Maximum cell mass of *M. gitamensis* BSH-3^T^ and L-asparaginase production was observed with glucose as a carbon source, whereas, low growth and L-asparaginase production was observed with starch followed by lactose and arabinose (Table 2). Among six different nitrogen sources used, maximum growth and L-asparaginase production was observed with yeast extract followed by soybean meal and beef extract. Low cell mass and L-asparaginase production was observed in the medium containing urea (Table 3). The optimum growth and L-asparaginase production was observed at pH=8.0 and beyond this there is a sudden decrease in growth and L-asparaginase production (Figure 1). This indicates that *M. gitamensis* BSH-3^T^ is a slightly alkalophilic organism. Figure 2 shows the optimum growth and L-asparaginase production at 40 °*C* and beyond optimal temperature, growth and L-asparaginase production was less. There was a sharp increase in the growth of *M. gitamensis* BSH-3^T^ and L-asparaginase production from 12–36 *hr* of incubation and gradual decrease up to 84 *hr* of incubation (Figure 3). This clearly showed that both growth and L-asparaginase production sharply decreased to 50% after 48 *hr* of incubation.

**Table 2.** Effect of carbon source on the growth of *M. gitamensis* BSH-3^T^ and production of L-asparaginase

**Carbon source**	**Enzyme activity (*IU*)**	**Cell mass (*mg/ml*)**
**Arabinose**	98.6±1.2	0.78±0.044
**Fructose**	108.4±0.9	0.85±0.041
**Glucose**	124.8±2.1	1.17±0.038
**Lactose**	98.1±0.8	0.67±0.042
**Maltose**	104.8±1.1	0.75±0.021
**Starch**	81.4±1.2	0.35±0.020
**Sucrose**	102.2±2.3	0.68±0.032

**Table 3.** Effect of nitrogen source on *M. gitamensis* BSH-3T

**Nitrogen source**	**Enzyme activity (*IU*)**	**Cell mass (*mg/ml*)**
**Beef extract**	160.8±3.4	0.82±0.013
**Malt extract**	152.4±4.2	0.76±0.023
**Peptone**	158.2±5.2	0.78±0.031
**Tryptone**	156.4±6.7	0.74±0.035
**Urea**	123.2±3.4	0.43±0.024
**Yeast extract**	220.2±5.2	1.40±0.034

### Central Composite Design based optimization (CCD)

The production of L-asparaginase by *M. gitamensis* BSH-3 was maximized by optimization of the medium components through Central Composite Design (CCD) of response surface methodology. Thirty-two experiments were conducted in triplicate with different concentrations of wheat bran, yeast extract and different pH, temperature and incubation time (Table 4). The response obtained from experiments of Central Composite Design (Table 5) were calculated with second order polynomial multiple regression.

**Table 4.** Experimental design of five variables with coded values for the production of L-asparaginase

**Run order**	**A**	**B**	**C**	**D**	**E**	**Enzyme specific Activity (*IU*)**	
						**Experimental**	**Predicted**
**1**	+1	−1	+1	+1	−1	205	211.9
**2**	0	0	+1	0	0	279.8	263.4
**3**	−1	+1	+1	−1	+1	108.3	105.4
**4**	0	0	−1	0	0	241.6	257.9
**5**	0	0	0	0	+1	295.6	288.5
**6**	0	0	0	0	0	263.9	268.4
**7**	0	0	0	0	0	278.3	268.4
**8**	+1	−1	−1	+1	+1	216.9	221.5
**9**	+1	+1	+1	−1	−1	231.8	232.1
**10**	−1	0	0	0	0	119.9	139.6
**11**	0	0	0	0	−1	282.1	289.1
**12**	−1	−1	+1	+1	+1	103.2	106.9
**13**	−1	+1	−1	−1	−1	111.2	102.4
**14**	0	0	0	0	0	265.1	268.4
**15**	−1	+1	+1	+1	−1	108.3	107.8
**16**	+1	+1	+1	+1	+1	215	221.2
**17**	−1	−1	−1	−1	+1	110.2	105.6
**18**	−1	−1	+1	−1	−1	116.4	114.2
**19**	+1	−1	−1	−1	−1	218.5	217.1
**20**	+1	0	0	0	0	269.1	249.2
**21**	0	0	0	−1	0	227	243.8
**22**	0	+1	0	0	0	237.8	247.6
**23**	0	0	0	+1	0	258.9	242.1
**24**	−1	+1	−1	+1	+1	109.3	106.5
**25**	−1	−1	−1	+1	−1	110.4	108.3
**26**	+1	+1	−1	+1	−1	204	204.4
**27**	+1	−1	+1	−1	+1	213.6	218.1
**28**	0	0	0	0	0	269.4	268.4
**29**	0	0	0	0	0	271.9	268.4
**30**	0	−1	0	0	0	259.5	249.7
**31**	0	0	0	0	0	261.7	268.4
**32**	+1	+1	−1	−1	+1	209.6	207.5

Enzyme specific activity (*IU*)=28554.2+808.023 *A+203.875 *B+035.073 *C+527.393 *D-1949.05 *E-73.9135 *A^2^-19.7035 *B^2^-7.71355 *C^2^-25.4035 *D^2^+20.4305 *E^2^+0.593750 *AB+1.33125 *AC-1.10025 *AD+0.093750 *AE+2.94375 *BC-0.0087500 *BD-0.418750 *BE-1.85025 *CD-1.45025 *CE+3.30025 *DE.

**Table 5.** ANOVA analysis of RSM model for enzymatic production individual factors

**Source**	**DF ^a^**	**Seq SS ^b^**	**Adj MS ^c^**	**F**	**p**
**A**	1	54044	54044	225.43	0.014
**B**	1	19	19	0.08	0.785
**C**	1	137	137	0.57	0.405
**D**	1	14	14	0.00	0.817
**E**	1	2	2	0.01	0.929
**A^2^**	1	13443	13443	50.08	0.002
**B^2^**	1	2219	2219	4.01	0.017
**C^2^**	1	243	243	0.01	0.021
**E^2^**	1	1028	1028	4.29	0.043
**AB**	1	0	0	0.19	0.881
**AC**	1	28	28	0.02	0.980
**AD**	1	20	20	0.12	0.737
**AE**	1	8	8	0.08	0.801
**BC**	1	139	139	0.03	0.541
**BD**	1	2	2	0.58	0.101
**BE**	1	3	3	0.02	0.010
**CD**	1	55	55	0.01	0.020
**CE**	1	34	34	0.23	0.201
**DE**	1	175	175	0.14	0.180

Analysis of variance (ANOVA) was performed on data collected from experiments as shown in table 6. The coefficient of determination R^2^ showed the appropriateness of the adequate model. The above results were analyzed and calculated and determination of coefficient (*R*^2^) was found to be 0.9809 indicating that the statistical model can explain 98.09% of variability in the response and only 1.91% of the total variations were not explained by the model. In the present study, for the production of L-asparaginase, the adjusted *R*^2^ value (0.9401) was less than the *R*^2^ value (0.9809). The adjusted *R*^2^ may be distinctly smaller than the *R*^2^ with a low value of the coefficient of variation which indicates good precision and reliability of the study.

**Table 6.** ANOVA analysis of RSM model for production of L-asparaginase by *Myroides* sp BSH-3

**Source**	**DF ^a^**	**Seq SS ^b^**	**Adj SS ^b^**	**Adj MS ^c^**	**F**	**p**
**Regression**	20	135174	135174	0758.7	28.19	<0.001
**Linear**	5	54215	54215	10843.1	45.23	0.001
**Square**	5	80492	80492	10098.3	07.15	<0.001
**Interaction**	10	407	407	40.7	0.19	0.004
**Residual error**	11	2037	2037	239.7	-	-
**Lack of fit**	0	2450	2450	408.3	10.90	0.001
**Pure error**	5	187	187	37.5	-	-
**Total**	31	137811				
R^2^= 98.09%, R^2^=94.01%						

Note:

a: Degree of freedom;

b: Sum of Squares;

c: Mean Squares.

The interaction of the L-asparaginase activity and independent variables was demonstrated by three dimensional response surface diagrams. In each 3D curve, effect of two factors on enzyme activity was shown, maintaining other variables constant at level zero.

### Surface plots

**Figure 4.**
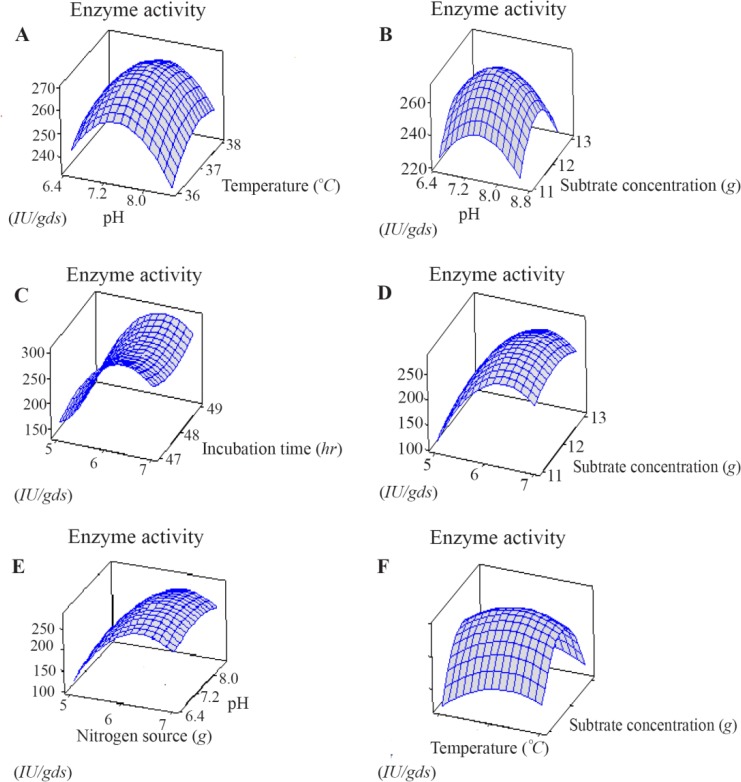
Three dimensional response surface plot for the effect of (A) pH, temperature (B) pH, Substrate concentration (C) Nitrogen source, Incubation time (D) Nitrogen source, substrate concentration (E) Nitrogen source, pH (F) Temperature, substrate concentration.

Surface plots are generally the graphical representation of the regression equation for identifying the optimal levels of each parameter for attaining the maximum response (L-asparaginase) production. The response surface plots of L-asparaginase were presented in figure 4 (A-F). The pair-wise interaction generated from 3D graphs of the five parameters explains the role of different parameters which affect the production of L-asparaginase. Figure 4F shows that 12 *g* of wheat bran is required for maximum specific activity (270 *IU*) was observed at the mid value of substrate amount (12 *g*). Figures 4A and B show the effect of pH on L-asparaginase production in combination with substrate concentration (wheat bran) and temperature. There is increase in L-asparaginase production with increase in pH and the maximum production was at pH=7.5. In previous studies, Mohammad *et al* and Rajesh *et al* have reported that the commercial production of L-asparaginase by submerged fermentation using different bacterial species required optimum pH between around pH=8.0 19,20. The L-asparaginase activity steadily increased with increase in temperature and reached a maximum activity (270 *IU*) at the optimal value at 37°*C*. The same effect of temperature on the growth and L-asparaginase production by *Cylindrocarpon obtusisporum* MB-10, *Pseudomonas stutzeri* MB-405, *Bacillus* sp, have been observed 21,22. Figure 4C shows that 6 gram of yeast extract is required for maximum production of L-asparaginase (295 *IU*) and it remains constant with variation in incubation time. Maximum L-asparaginase production using yeast extract as a sole source of nitrogen in *Fusarium equiseti* was reported 23.

### Purification of crude enzyme

**Figure 5.**
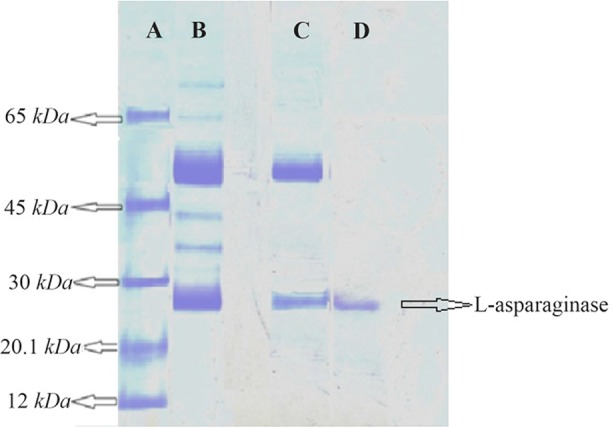
SDS-PAGE of L-asparaginase. Lane A) Protein molecular markers. Lane B) Crude enzyme. Lane C) Ammonium sulphate precipitation (20–40%). Lane D) Sephadex G-200 chromatography fraction.

The enzyme was salted out in 20–40% ammonium sulphate fraction and this fraction was further purified by gel filtration on sephadex G-200 gel filtration column. The L-asparaginase was eluted as a single peak infraction. These fractions were pooled, lyophilized and used for further studies. SDS-PAGE analysis of the purified L-asparaginase showed a single band (Figure 5). L-asparaginase was purified to apparent homogeneity with molecular mass of 26 *kDa*.

## Discussion

Production of enzymes by microorganism depends on many parameters like nutrients, salt concentration, pH and temperature. In fact, the composition of culture medium is closely associated with the metabolic capacities of the producing strain and it significantly influences the biosynthesis of enzymes 24. The concept of medium optimization for enzyme production involves the exploitation of medium components and cultural condition to obtain the desired product in a cost effective manner.

Therefore, selection of right production medium is necessary for optimal production of preferred enzyme 25. As the characteristics of the microorganisms are modified continuously with time, the fermentation process and media should also be changed for the enhanced production of enzyme.

The fermentative production of enzymes is influenced by various factors such as the choice of microorganism selected, choice of raw material used, inoculum level, temperature, pH, aeration, agitation, duration of fermentation, and type of fermentation, precursors used in the fermentation medium and enzyme concentration produced during fermentation 26. Hence, the fermentation process and fermentation media have been continuously updated to obtain better yields of enzyme production.

To find the optimized parameters, response surface methodology is a suitable mathematical and statistical tool; it can be helpful to find experimental design for illuminating the relations between different parameters. Recently, RSM was used extensively to optimize fermentation parameters 27. The current study reveals that wheat bran had impact on the production of L-asparaginase production. Wheat bran contains approximately 62% carbohydrate, 18% protein and 5% fat and it is a relatively complete source of nutrients for microbes 28–30. The use of a perfect agro-residual based substrate for enhancing enzyme production in a solid-state fermentation process mostly depends upon its easier degradation into nutrients and its utilization by the microorganism to produce the targeted metabolite. The present investigation revealed that L-asparaginase production varied with agro-residual wheat bran substrate. The production of L-asparaginase by microorganisms is highly dependent upon medium pH as it plays a crucial role in transportation of different components across the cell membrane and in organizing the metabolic behavior of the cell 31. Results showed that enzyme production was increased with pH achieving an optimum level at pH=7.5. Further production of the enzyme decreased subsequently at higher pH values attaining activity up to pH=10.0.

## Conclusion

Central Composite Design based response surface methodology for multi parameter optimization is useful for investigating the optimal conditions. The present investigation optimizes different process parameters namely, incubation time, pH, temperature, nitrogen source and substrate concentration for the enhancement of L-asparagianse production by *M. gitamensis.* The coefficient of determination (*R^2^*) values for all parameters showed a goodness of fit between the predicted model and an experimental data with a confidence level of 95%. Analysis of different cultural conditions (incubation time, pH, temperature, nitrogen source and substrate concentration) are required for the commercial scale production of L-asparaginase enzyme. The results obtained in the current investigation were fitted well with an experimental data and the predicted models. The currently adopted approach can be applied for any enzyme production on commercial scale. Optimization of the solid substrate was a very important measure to increase enzyme activity and realize industrial production of L-asparaginase. The process of L-asparaginase production in laboratory scale may have the scale-up potential.
